# Daily update of motor predictions by physical activity

**DOI:** 10.1038/srep17933

**Published:** 2015-12-03

**Authors:** Nicolas Gueugneau, Nicolas Schweighofer, Charalambos Papaxanthis

**Affiliations:** 1 CAPS (Cognition, Action et Plasticité, Sensorimotrice), INSERM (Institut National de la Santé et de la Recherche Médicale) UMR1093, Université Bourgogne Franche-Comté, F-21000 Dijon, France; 2 Biokinesiology and Physical Therapy, University of Southern California, Los Angeles, CA 90089-9006, USA

## Abstract

Motor prediction, i.e., the ability to predict the sensory consequences of motor commands, is critical for adapted motor behavior. Like speed or force, the accuracy of motor prediction varies in a 24-hour basis. Although the prevailing view is that basic biological markers regulate this circadian modulation, behavioral factors such as physical activity, itself modulated by the alternation of night and day, can also regulate motor prediction. Here, we propose that physical activity updates motor prediction on a daily basis. We tested our hypothesis by up- and down-regulating physical activity via arm-immobilization and high-intensity training, respectively. Motor prediction was assessed by measuring the timing differences between actual and mental arm movements. Results show that although mental movement time was modulated during the day when the arm was unconstrained, it remained constant when the arm was immobilized. Additionally, increase of physical activity, via release from immobilization or intense bout of training, significantly reduced mental movement time. Finally, mental and actual times were similar in the afternoon in the unconstrained condition, indicating that predicted and actual movements match after sufficient amount of physical activity. Our study supports the view that physical activity calibrates motor predictions on a daily basis.

An important feature of motor behavior is our ability to predict the consequences of our actions before sensory feedback is available. Evidence support the hypothesis that motor prediction is generated by internal forward models, which are neural networks that mimic the causal flow of the physical process by predicting the future sensorimotor state (e.g., position, velocity) given the efferent copy of the motor command and the current state^1^,^2^. Motor prediction via forward models is useful in anticipation and cancellation of the sensory consequences of movement, as well as in mental practice^2^,^3^. In addition, forward models are crucial in production of quick and accurate movements: by replacing the delayed consequences of our movements derived from sensory inputs, the predictions from the forward models can be used in high-gain internal feedback loops with short delays^4^,^5^. Forward models are not fixed entities, however, but must continuously adapt to internal (fatigue, growth, etc.) or external (interaction with novel objects, etc.) perturbations at difference time scales. In theory, such adaptation is achieved via “self-supervised learning”^6^, a process that minimizes prediction errors, namely the errors between the output from the forward model and the sensory outcome of the motor command. In such a scheme, movement is vital for learning, adapting, and maintaining accurate internal forward models. For instance, it is well established that arm immobilization severely affects motor performance^7^,^8^.

Surprisingly, like movement speed^9^ or muscular force^10^, motor predictions of well-learned movements, such as walking, pointing or writing, are dramatically modulated during the day^11^,^12^,^13^. What is the cause of such a circadian modulation of motor prediction? While basic biological factors^14^,^15^ can influence this circadian fluctuation, sensorimotor activities may also have a potential role and should be explored. Here, we propose that daily movements, or basic daily activities, strongly regulate motor prediction. The reason for such a relationship is straightforward: as movements are necessary to improve motor prediction via self-supervised learning and at the same time our daily activity is itself circadian (the alternation of night and day is accompanied by periods of rest and physical activity), daily movements should underlie the modulation of motor prediction. To test this hypothesis, we manipulated physical activity by decreasing it via arm immobilization and increasing it either via release from immobilization or via bouts of high-intensity motor training. To measure motor prediction, we used motor imagery, in which subjects perform movements mentally, without actual execution^16^,^17^. Mental and actual movements engage similar motor areas^18^, and forward models are believed to be involved in mental movement simulation^1^,^2^.

A key point here is the fact that the temporal features of mental movements emerge from sensorimotor predictions of the forward models^1^,^19^,^20^. During motor imagery, motor commands are prepared, but are blocked before reaching the muscle level; i.e., no movement occurs. However, a copy of these motor commands, the efference copy, is still available to the forward model, which predicts the future sensorimotor states of the arm and thus provides temporal information that are very similar to that of actual movements. Data from healthy subjects^16^,^17^, from patients^19^,^21^, as well as from developmental^22^,^23^ and learning studies^24^, indicate that the tight temporal relation between mental and actual movements is a hallmark of the normally developed motor system, and constitute a solid set of evidence indicating that the timing of mental movements arises from forward models. For instance, when asked to mentally imagine to reach to targets to the right of the left of the workspace, the modulation of mental movement times with respect to target location show an excellent correspondence to actual movement times, which have been explained on the basis of the arm’s dynamics^25^,^26^. Therefore, in this paper, the accuracy of motor prediction is estimated by the difference between actual movement time and mental movement time^16^,^17^,^19^.

## Results

### Physical activity modulates mental movement times during the day

**Figure 1:**
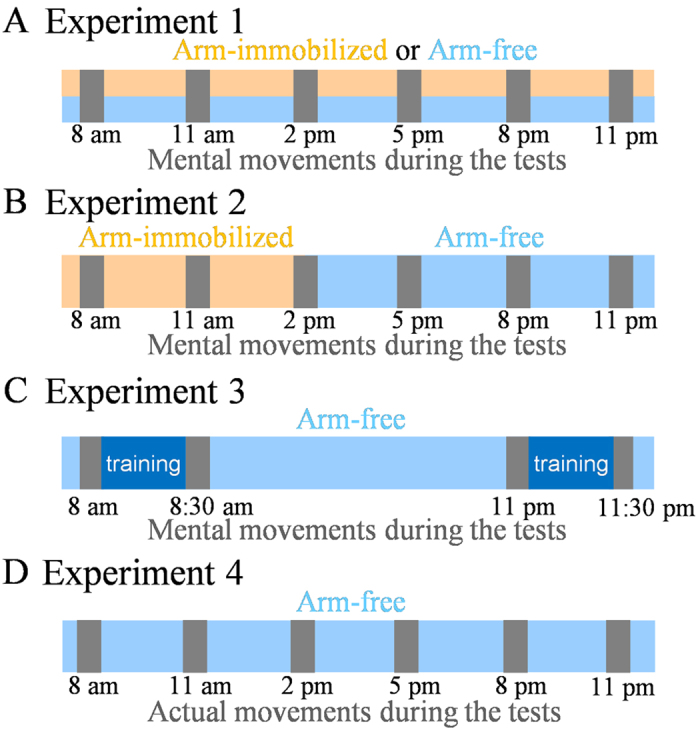
Experimental protocol. (**A–D**) illustrate our four main experiments. Each vertical grey rectangle indicates a testing session where actual or imagined movements were recorded. Control experiments and details for each experiment are provided in the Methods section.

In the first experiment, we tested the core hypothesis of our study; namely, whether motor prediction – assessed by mental movement time – was modulated during the day under the influence of physical activity. Ten participants (group 1) carried out mental pointing movements 6 times during the day (6 tests: 8 a.m., 11 a.m., 2 p.m., 5 p.m., 8 p.m., and 11 p.m.) in two conditions: arm-free and arm-immobilized (Fig. 1A). At each test, participants performed 10 mental trials, each trial included 10 consecutive mental pointing movements (i.e., 100 mental movements per test), between two small targets (radius 0.5) spaced 20 cm apart, with the instructions to move mentally as accurately and as fast as possible (kinesthetic movement representation, as in^16^,^17^).

**Figure 2:**
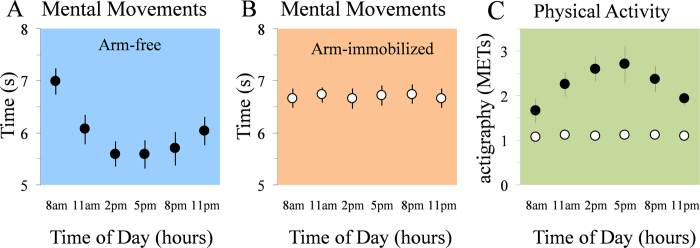
(**A**) Experiment 1: mental movement times in the arm-free condition. Participants’ mean and SE (standard error) of mental movement times in the 6 tests. There was a large modulation of mental movement time during the day, as it progressively decreased from 8 a.m. to 5 p.m. and progressively increased from 8 p.m. to 11 p.m. (**B**) Experiment 1: mental movement times in the arm-immobilized condition. Participants’ mean and SE (standard error) of mental movement times in the 6 tests. It can be observed that mental movement time remained stable during the day. (**C**) Mean and SE of the amount of physical activity during the day recorded by an actigraph from subjects’ right arm. In the arm-free condition (black-filled symbols), physical activity showed a large daily modulation, while it remained stable during the day in the arm-immobilized condition (black-opened symbols).

In the arm-free condition, during which participants were free to use their arms in their normal daily activities, mental movement time showed a large modulation during the day (cosinor analysis: P < 0.001, R^2^ = 0.95; Fig. 2A). The average amplitude of the fitted cosine curve was 1.59 ± 0.17 s (>0, t(9) = 9.07, P < 0.0001), with faster mental time at 4.54 ± 0.12 p.m. rANOVA showed a main effect of time (F_5,45_ = 25.80, P < 0.0001; partial η^2^ = 0.74). Post hoc analysis showed significant differences between: a) 8 a.m. vs. all the others times (in all cases, P < 0.001), b) 11 a.m. vs. 2 p.m. (P = 0.03), c) 11 a.m. vs. 5 p.m. (P = 0.02), d) 5 p.m. versus 11 p.m. (P = 0.04). There are two potential modulators for the variation of mental movement time during the day: physical activity and the simple passage of time. As we could not act on the passage of time, we controlled for the effect of physical activity by immobilizing the participants’ dominant arm with a sling from a 7 a.m. to 11 p.m. In this condition, the cosinor analysis failed to detect any variation of mental movement time during the day (P > 0.1; Fig. 2B) showing that physical activity influences motor prediction daily. rANOVA showed that mental movement time was stable during the day (F_5,45_ = 0.44, P = 0.81; 95% of Interval Confidence: 6.2 s–7.1 s at 8 a.m., 6.3 s–7.2 s at 11 a.m., 6.3 s–7.2 s at 2 p.m.; 6.2 s–7.1 s at 5 p.m., 6.2 s–7.1 s at 8 p.m., and 6.4 s–7.1 s at 11 p.m.). To evaluate the amount of physical activity during the day, participants wore an actigraph on their right wrist from 7 a.m. to 11 p.m. In the arm-free condition (Fig. 2C, black symbols), there was a large daily modulation of physical activity (cosinor analysis; P < 0.001, R^2^ = 0.71). The amplitude of fitted cosine curve was 1.44 ± 0.3 s (>0, t(9) = 4.5, P = 0.001) with a peak at 3.45 ± 0.27 p.m. Using all data points from all subjects, physical activity (obtained via actigraphy) was negatively correlated with mental movement time (r = −0.47; t(60) = −4; P < 0.001). On the contrary, in the arm-immobilized condition (Fig. 2C, white symbols), physical activity was stable during the day (cosinor analysis; P > 0.1) and significantly lower compared to the arm-free condition (respectively: 1.10 ± 0.01 METs and 2.35 ± 0.04 METs; P < 0.001, t(9) = 10.99).

**Figure 3:**
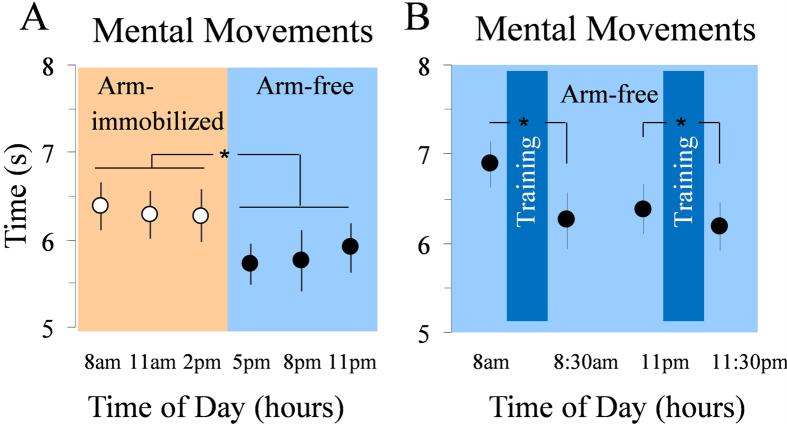
(**A**) Experiment 2: mental movement times in the arm-immobilized condition until the mid-day. Participants’ mean and SE of mental movement times in the 6 tests (orange panel: arm-free condition; blue panel: arm-immobilized condition). While mental movement time remained stable until 2 p.m., the removal of the splint at 2 p.m. led to significantly reduction of mental movement time. (**B**) Experiment 3: mental movement times after physical training. The training session consisted of using a scissor for cutting geometrical figures (10 min), writing a text (10 min), and playing with a small ball (10 min). Participants’ mean and SE of mental movement times in the pre-test and post-test sessions. Mental time significantly decreased in the post-training sessions compared with the pre-training sessions.

The above findings indicate that the absence of physical activity severely weakens the daily modulation of mental movement times. In two complementary experiments, we tested whether amplified physical activity also affects mental movement time during the day. In Experiment 2, the dominant arm of ten new participants (group 2) was immobilized with a sling in the first part of the day (from 7 a.m. to 2 p.m.) and was then let free to move (2 p.m. to 11 p.m.; see Fig. 1B and methods for details). In light of the above results, we expected mental movement time to be stable during immobilization (as in Fig. 2B) and then to decrease with physical activity (as in Fig. 2A). Indeed, removal of the splint led to a significant reduction of mental movement time (Fig. 3A). ANOVA showed a main effect of time (F_5,45_ = 14.84; P < 0.0001; partial η^2^ = 0.63). Post hoc analysis showed significant differences between 8 a.m., 11 a.m., and 2 p.m. versus 5 p.m., 8 p.m., and 11 p.m. (in all, P < 0.001). In Experiment 3, ten new participants (group 3) performed mental movements before and after an intensive 30-minutes physical training session administrated in the morning at 8 a.m. and in the evening at 11 p.m. (Fig. 1C). The added physical activity decreased mental movement time in the morning (t(9) = 4.79; P < 0.0001) and prevented its increase in the evening (post-training durations are smaller than pre-training durations t(9) = 4.02; P = 0.003) as shown in Fig. 3B.

In all above experiments, we verified that arm muscles remained silent during mental arm movements. RMS values of all muscles during mental trials were very low (mean individual values ranged between 4 μV and 11 μV) and similar (in all cases, t(9) < 1.7, P > 0.1) to those obtained when the same muscles were totally relaxed (mean values ranged between 4 μV and 9 μV).

### Physical activity modulates the temporal similarity between actual and mental movements during the day

**Figure 4:**
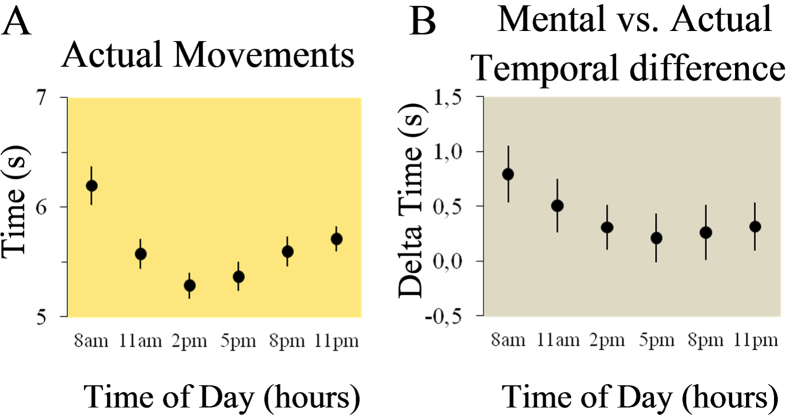
(**A**) Experiment 4: actual movement times in the arm-free condition. Participants’ mean and SE of actual movement times in the 6 tests. Actual movement time was modulated during the day as it progressively decreased from 8 a.m. to 2 p.m. and then progressively increased from 5 p.m. to 11 p.m. (**B**) Temporal difference between metntal and actuall movements. Participants’ mean and SE (standard error) of the temporal difference between mental and actual movement in the 6 tests. Positive values indicate greater times for mental movements.

We then investigated the accuracy of motor prediction by comparing the daily modulation of actual movement times (Experiment 4) with the daily modulation of mental movement times (Experiment 1). Ten participants (the same as in Experiment 1, group 1) carried out arm pointing movements every 3 hours (8 a.m., 11 a.m., 2 p.m., 5 p.m., 8 p.m., and 11 p.m.) in an arm-free condition (Fig. 1D). Participants performed 10 actual trials, each trial included 10 consecutive pointing, between two small targets (radius 0.5) spaced 20 cm apart, with the instructions to move as accurately and as fast as possible. Actual movement time significantly varied during the day (cosinor analysis, P < 0.001, R^2^=0.94; Fig. 4A). The amplitude of the fitted cosine curve was 1.17 s ± 0.09 (>0, P < 0.0001, t(9) = 12.71), with faster times at 4.17 ± 0.32 p.m. rANOVA showed a main effect of time (F_5,45_ = 15.56, P < 0.0001; partial η^2^ = 0.6). Post hoc analysis showed significant differences between: a) 8 a.m. vs. all the others times (in all, P < 0.001), b) 2 p.m. vs. 11 p.m. (P = 0.007), c) 5 p.m. vs. 11 p.m. (P = 0.04). The variation in movement time across the test cannot be attributed to a motor strategy that shifted between accuracy and speed through the day, because the total number of errors, i.e., the total times that participants pointed outside the targets, was constant and not different through the 6 tests (respectively, 38, 39, 40, 39, 40 and 37 total errors, ANOVA Friedman, χ^2^ = 1.93; P = 0.85).

We then compared mental and actual arm movement durations in the arm-free condition. rANOVA revealed an interaction effect of *time* and *movement* (F_5,45_ = 3.51, P = 0.009; partial η^2^ = 0.28). Mental movement time was longer than actual movement time (compare Figs 2A and 4A, and see Fig. 4B) at 8 a.m. (P < 0.001), at 11 a.m. (P = 0.006), and at 11 p.m. (P = 0.02), but not different at the other times of the day (in all, P > 0.4). This finding indicates that temporal similarity between actual and mental movements is progressively acquired and progressively lost during the day (Fig. 4B). As expected, the difference between actual and mental movement times was negatively correlated with physical activity (i.e., actigraphy during arm free) during the day (r = −0.45; t(60) = 3.6; P < 0.001): the greater the activity the smaller the difference between actual and mental movement times.

### Control experiments

**Figure 5:**
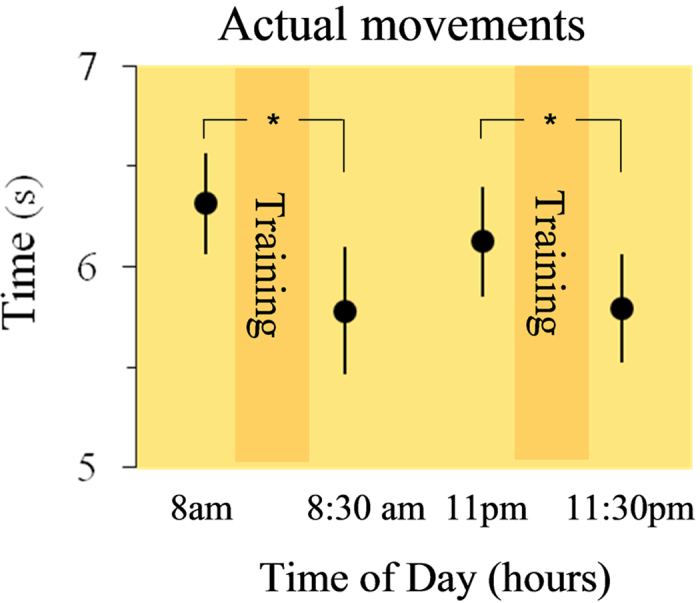
Actual movement times after physical training (Control Experiment 1). The training session consisted of using a scissor for cutting geometrical figures (10 min), writing a text (10 min), and playing with a small ball (10 min). Participants’ mean and SE of actual movement times in the pre-test and post-test sessions. Actual time significantly decreased in the post-training sessions compared with the pre-training sessions.

To further examine whether a physical training session positively influences actual movement times, we also ran a control experiment (control experiment 1) in which participants (the same as in Experiment 2) carried out a morning (8 a.m.) and an evening (11 p.m.) 30-min motor training session, which was similar with the training session used for mental movements (see Methods section). The added physical activity had positive effects on actual movement times (Fig. 5), similar to those on mental movement times. Precisely, the training session reduced movement time the morning (−8.46%, t(9) = 3.21, P = 0.01) and the evening (−5.47%, t(9) = 2.72, P = 0.02) between the pre-training and the post-training sessions.

In a second control experiment, we verified that the modulation of mental and actual times in the arm-free condition during the day was not specific to the fixed order (i.e., from 8 a.m. to 11 p.m.) that participants carried out the tests (see control experiment in S1).

## Discussion

We examined how physical activity during the day influences the modulation of motor prediction. We measured motor prediction by asking subjects to mentally simulate arm movements, a processes which involves forward internal models^1^,^2^ and engage similar motor areas as actual movement production^18^. We showed that: (i) when the arm was free to move, mental movement time was highly modulated during the day; (ii) when physical activity was prevented during the day by means of arm-immobilization, mental movement time was stable during the day; (iii) increase of physical activity through physical training significantly reduced mental and actual movement time; and (iv) mental and actual times were similar in the afternoon in the free arm condition, indicating that predicted and actual states match after sufficient amount of physical activity. These results support the view that physical activity calibrates internal model predictions daily. Because the amount of physical activity varies in a 24-hour rhythm, motor prediction updating follows a similar circadian variation. Reduction of physical activity during the day, via immobilization of the arm during 16 h from 8 a.m., did not allow sufficient movements to retrain the forward model and therefore mental movement time did not improve during the day. The negative effects of immobilization are rapidly reversible, however, because physical activity after immobilization or intensive training can improve motor prediction.

Daily variations of motor prediction may be due to plastic changes operating either at the level of the forward model itself in cerebellar and parietal networks^20^,^27^,^28^, or at the level of the motor command, which provide the inputs to the forward model via efferent copy. Changes in motor commands via activity-dependent “use-dependent learning” are probable in our experiments, because repetition of movements induce rapid plastic changes into the motor cortex^29^,^30^ and improve arm kinematics^31^,^32^,^33^,^34^. In addition, short-term limb immobilization (<24 hours), induces synaptic remodeling in sensory and motor cortical areas^35^, reduces corticospinal excitability^36^,^37^,^38^, and affects kinematic parameters^7^,^8^. Is it thus possible that changes in the motor command due to use-dependent learning, brought about by use of the arm through the day, solely accounts for the modulation of mental movement times in our study? We believe that this is unlikely, because self-supervised learning changes the mapping between motor commands and the sensory consequences by improving predictions^28^; in contrast, use-dependent learning is an unsupervised learning process that does not improve predictions. Therefore, changes in mental movement time during the day, and its convergence toward the actual movement the afternoon, reflect changes in forward model predictions, and suggest a strong self-supervised learning component. Worsening of mental performance in the evening may be due, however, to a use-dependent learning mechanism or to “unadapting” of the forward models via forgetting^39^, or both. Note that such a phenomenon was not observed in the arm immobilization condition, in which mental movement times remained constant during the day. It is likely that the effect of immobilization in our experiment was not strong enough to deteriorate mental or actual movement times from the morning (8 a.m.). It is plausible that several days of immobilization could severely affect forward model and thus increase mental and actual movement times form the early morning until the evening. In such a case, more physical activity that just moving the arm during the day would be necessary to restore motor performance.

In contrast to motor adaptation paradigms that deal with the acquisition of new internal models of specific “tools”, we investigated here the updating of existing forward models of the arm. Computational models have suggested that acquisition of internal models occurs across multiple time-scales, from seconds to days^40^,^41^. We demonstrated here an intermediate time-scale, in which forward models of the arm are calibrated on a daily basis via physical activity. Such an intermediate scale may arise because calibrating forward models of the arm is a mighty task due to the high complexity of non-linear dynamics and large number of degrees of freedom. Note, however, that a high-intensity motor training bout can accelerate updating and tuning of the forward model by providing a higher rate of training movements.

In summary, our results suggest that physical activity calibrates well-acquired predictive internal models through the process of self-supervised learning^6^. This expands previous findings showing that physical activity maintains the excitability and synaptic plasticity of the cortical motor network^38^. In the morning, because of disuse-dependent forgetting during the night and during low periods of activity, the forward model is not well-tuned to the characteristics of the arm. The forward model is not degraded to the point of non-functioning, nonetheless: as the general rule a poor model is better than no model at all^42^. However, well-tuned motor predictions are ecologically very useful, notably for fast feedback control. Thus, the enlightened hunter or warrior knew that fighting early in the morning or late at night was not optimal for survival.

## Methods

### Participants

Forty healthy adults gave their written consent to participate in this study. All were synchronized with a normal diurnal activity (7 a.m. to 12 p.m.) alternating with a nocturnal rest. Their chronotype^43^ was moderate morning type (n = 12) or neither type (n = 28). Body temperature of all the participants showed a regular circadian variation (P < 0.001, R^2^ = 0.98, maximum at 3.40 ± 0.4 p.m. average amplitude of the fitted cosine curve = 0.49° ± 0.05 C). All participants were right-handed^44^ and good imagers^45^. One day before and the day of the experiments, participants were not engaged in any physical activity in order to prevent fatigue. Informed consent was obtained from all subjects. All experiments were conducted in accordance to the principles in the Declaration of Helsinki and were approved by the regional ethics committee of Burgundy (AEC/B90097-40).

### Experimental design

In *Experiment 1* we investigated the effects of physical activity on the modulation of mental movement time. Ten participants (group 1: 4 females; mean age = 28.19 ± 3.04 years; MIQ-R score = 43.8 ± 2.7; Handedness score = 0.86 ± 0.04) carried out imagined arm movements every 3 hours (6 tests: 8 a.m., 11 a.m., 2 p.m., 5 p.m., 8 p.m., 11 p.m.) in two conditions: *arm-free*, participants were free to use their arms during the day without any specific instructions on arm use; *arm-immobilized*, the participants’ right arm was immobilized by means of an arm sling from 7 a.m. to 11 p.m. The sling was removed just prior to the tests and was replaced immediately after by the experimenter.

In *Experiment 2* we studied the effects of release from immobilization at mid-day on mental movement time. We tested ten new participants (group 2: 5 females, mean age = 26.82 ± 2.72 years; MIQ-R score = 45.1 ± 4.7; Handedness score = 0.87 ± 0.04). Their right arm was immobilized with the same procedure described above (Experiment 1) from 7 a.m. to 2 p.m., and then it was let free until 11 p.m.

In *Experiment 3* we examined whether physical training can improve mental movement time. Ten new participants (group 3: 4 females; mean age = 27.02 ± 4.13 years; MIQ-R score = 44.3 ± 2.5; Handedness score = 0.85 ± 0.04) performed mental arm movements the morning and the evening. They accomplished 10 mental trials (pre-training session; at 8 a.m. or at 11 p.m.), immediately after they received a 30 min physical training, and subsequently they re-accomplished 10 mental trials (post-training session; at 8:30 a.m. or at 11:30 p.m.). The training session consisted of using a scissor for cutting geometrical figures (10 min), writing a text (10 min), and playing with a small ball (10 min). The very same experiment was also conducted with actual movement (*Control experiment 1*) where we examined whether physical training can improve actual movement time. The participants of experiment 2 were recruited and followed the protocol above mentioned of experiment 3 with the difference that subjects actually performed the motor task.

In *Experiment 4* we compared actual with mental movement times during the day. The participants (group 1) and the protocol were the same with the Experiment 1 with the difference that the participant carried out actual arm movements, instead of imagined arm movements, during the tests.

All the above experiments were carried out in different days. Subjects of group 1, who participated in Experiments 1 and 4, performed first the mental movements in the arm-free condition, one week later the mental movements in the arm-immobilized condition, and one-week later the actual movements in the arm-free condition. Subjects of the groups 2 and 3 carried out the experiment in one day. In all experiments, participants had a normal activity between the tests, such as walking, discussing, eating, working on the computer or reading, etc., with the exception that in the arm-immobilized condition they did not use the immobilized arm.

### Experimental protocol

In all experiments during the 6 daily tests, the participants were comfortably sited in front of a table. Two targets (radius 0.5 cm; inter-target distance: 20 cm) were drawn on a sheet of A4 format. The perpendicular distance of the targets from the participant’s chest was 20 cm, 10 cm below the xyphoid process. During actual movements (Experiment 4) participants were instructed to hold a pencil in their right hand, and to point between the two targets *as accurately and fast as possible*. Participants carried out 10 trials on each test and each trial consisted of 10 alternated pointing movements between the two targets (i.e., 100 movements per test). The trial started either from the left or the right target (the starting position was pseudo-random and counter-balanced). During mental movements (Experiments 1–3) participants kept their right arm relaxed on the table. Their right index finger was placed either on the left or the right target (the starting position was pseudo-random and counter-balanced). From this position, they were requested to imagine pointing between the two targets *as accurately and fast as possible*. We instructed participants to feel themselves performing the motor task in a first person perspective (motor or kinaesthetic imagery, as in^17^). None of the participants reported difficulties to generate motor images. Participants carried out 10 imagined trials one each test and each imagined trial consisted of 10 alternated imagined pointing movements between the two targets. This was necessary due to the short duration of a single movement and the coarse resolution of mental movement time measurements – thus, several movements are necessary to obtain valid and reliable measurements in motor imagery protocols^19^. In experiments necessitating arm immobilization, the experimenter passively displaced the participants’ arm on the table and then back in the sling for the immobilization.

### Time recording and statistical analysis

In all experiments, the time of mental and actual arm movements was recorded by means of an electronic stopwatch (temporal resolution 1 ms) that participants hold in their left arm. They started the stopwatch when they actually or mentally initiated the movement and they stopped it when they had actually or mentally accomplished it. This mental chronometry paradigm is known to provide reliable results (for example see^16^,^17^,^19^). Mean values and standard errors (SE) for each participant were calculated in each condition. All variables were normally distributed (test Shapiro-Wilk, P > 0.05) and statistical significance was accepted at P > 0.05.

We first evaluated whether there was a variation of movement time during the day by means of the population mean cosinor analysis^46^,^47^. This analysis combines the results from single cosinor analysis of each subject to produce an average curve describing the considered variable. The best-fit 24h period cosine curve was calculated for each condition (mental-arm-free, mental-arm-immobilized, actual-arm-free), using the following formula (equation 1):


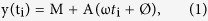


where M is the Mesor (i.e., the mean theoretical value around which the cosine model fluctuates), A is the Amplitude (=maximum value-minimum value), and Ø is the Acrophase (i.e., the time of the maximal theoretical value reached by the cosine curve referenced to local 00:00). Movement time variation during the day was validated when the cosine model fits well to the experimental values (goodness of fit determined by minimizing the sum of squares of the residuals from the analysis and by the coefficient of determination R^2^) and when significant amplitude was found. The latter is determined by the amplitude-test (F-test) that compares the variance accounted for the cosine approximation versus a straight-line fit to the time series data. We verified that the amplitudes of the cosine curves differed between experiments by means of two-tailed paired *t-tests.*

*Time* or *test* effects (6 tests: 8 a.m., 11 a.m., 2 p.m., 5 p.m. 8 p.m. and 11 p.m.) on movement time were tested by means of a one-way within-subjects repeated-measures analysis of variance (rANOVA) for each condition separately: mental movements in the arm-free and arm-immobilized conditions (Experiment 1), mental movements in the arm-immobilized condition until the mid-day (Experiment 2), and actual movements in the arm-free condition (Experiment 4). Comparison between mental movements in the arm-free condition (Experiment 1) and actual movements in arm-free condition (Experiment 4) was performed by means of a two-way rANOVA with *movement* (mental-actual) and *time* (8 a.m.–11 p.m.) as within-subjects factors. *Post hoc* differences were assessed by means of *Tukey* tests. The effects of physical training (Experiment 3 and control experiment 1) on mental or actual movement time were evaluated by means of two-tailed paired *t-tests* between pre-training and post-training sessions.

### Recording and statistical analysis of spatial accuracy

Spatial accuracy in Experiment 4 was measured by counting the number of times that participants did not point inside the targets. Spatial accuracy cannot be measured in Experiments 1–3 since participants performed mental movements. As this variable was not normally distributed (test Shapiro-Wilk, P < 0.05), *time* effects (8 a.m.–11 p.m.) upon spatial accuracy were evaluated by means of non-parametric analysis (ANOVA Friedman).

### EMG recoding and statistical analysis during mental movements

We tested for muscle activation during all mental trials (Experiments 1–3). EMG signals of the right arm muscles (anterior deltoid, posterior deltoid, biceps brachii, triceps brachii long head, flexor carpi radialis, extensor carpi ulnaris, and abductor pollicis brevis) were recorded by means of two silver-chloride surface electrodes of 10-mm diameter placed on the belly muscle with an inter-electrode distance (centre to centre) of 2 cm. EMG signals were also recorded at rest (5 trials of 6 s) before each test, by asking the participants to totally relax their muscles (rest condition). The reference electrode was placed on the inferior side of the left wrist. EMG signals were recorded at a frequency of 1000 Hz, band pass filtered (10–600 Hz) and stored for off-line analysis using BIOPAC^®^ software acquisition. Muscle activation during mental trial and rest was quantified by computing RMS (Root Mean Square) using the following formula (equation 2):


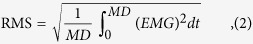


where MD is the movement duration.

To quantify muscle activation, we compared by mean of two-tailed *t-tests* for dependent samples (*Shapiro-Wilk W test*, P > 0.05) the RMS values during mental trials with RMS values recorded from the same muscles totally relaxed. Significance was accepted at P<0.05.

### Actigraphy

We recorded the level of physical activity of the right arm during the day (Experiments 1 and 2) via a 3D accelerometer system (32 Hz) included in a multisensor actigraph (InnerView Professional, SenseWear PRO Armband) placed on the right upper arm from 7:00 a.m., to 11 p.m. The actigraph computes the energy cost during movements in units called METs (metabolic equivalent). 1 MET is considered a resting metabolic rate obtained during quiet sitting, 0.9 during sleeping and 1.5 is considered as normal energy expenditure during deskwork^48^. We applied the population mean cosinor analysis (see above) on actigraph data to detect any circadian variation. Statistical difference of physical activity between arm-free and arm-immobilized conditions was assessed by paired two-tailed *t-tests* (*Shapiro-Wilk W test*, P > 0.05).

## Additional Information

**How to cite this article**: Gueugneau, N. *et al.* Daily update of motor predictions by physical activity. *Sci. Rep.* **5**, 17933; doi: 10.1038/srep17933 (2015).

## Supplementary Material

Supplementary Information
