# Supplementary material for: Daily update of motor predictions by physical activity
Source: Sci Rep. 2015 Dec 3;5:17933. doi: 10.1038/srep17933 (PMC4668580; doi:10.1038/srep17933)
Supplement: Supplementary Information [file srep17933-s1.pdf]

Daily update of motor predictions by physical activity

Nicolas Gueugneau[1,2*], Nicolas Schweighofer[3] and Charalambos Papaxanthis[1,2*]

1. Université de Bourgogne, UFR STAPS, F-21078 Dijon, France

2. Institut National de la Santé et de la Recherche Médicale (INSERM), Unité 1093, Cognition Action et Plasticité Sensorimotrice (CAPS), F-21078 Dijon, France.

3. Biokinesiology and Physical Therapy, University of Southern California, Los Angeles, CA 90089-9006, USA.

Supplemental Information

Control experiments

In this control experiment, we verified whether the order of performing the tests could influence the daily modulation of movement time. Indeed, in the main experiments, all participants performed the tests in a fixed order; i.e., from 8 a.m. to 11 p.m. Although, a protocol with a different schedule could be also proposed, for example, participants could accomplish the sessions in a random order during two or three days, such a protocol was not adapted for our immobilization condition, because we should immobilize participants' right arm for several days. Therefore, we examined that there were no qualitative differences between our arm-free protocol and a protocol in which the six tests would be accomplished in different days. Five new participants of (group 4, 2 females; mean age= 28.4±4.12 yrs; mean MIQ-R score= 44±4.2; mean handedness score= 0.85±0.03) carried out 6 tests of mental movements in a random order during 2 days and 6 tests of actual movements in a random order during 2 days one week latter. The tasks were similar to the main Experiment 1 and 4, respectively. We applied the population mean cosinor analysis, as described in the main text. Supplemental figure 1 (S 1) shows mean vales and SE across subjects. There was a large modulation of mental movement times, as revealed by the cosinor analysis: $P<0.001$, $R^2=0.89$, average amplitude of cosine curve=1.12±0.09 s (>0, $P<0.001$, $t(4)=12.07$). Similarly, actual movement times were modulated during the day: $P<0.01$, $R^2=0.91$, amplitude of cosine curve = 1.05 s ±0.10, (>0, $P<0.001$, $t(4)=11.12$). These findings indicate that the fixed or the random order of tests did not influence the time variation during the day.

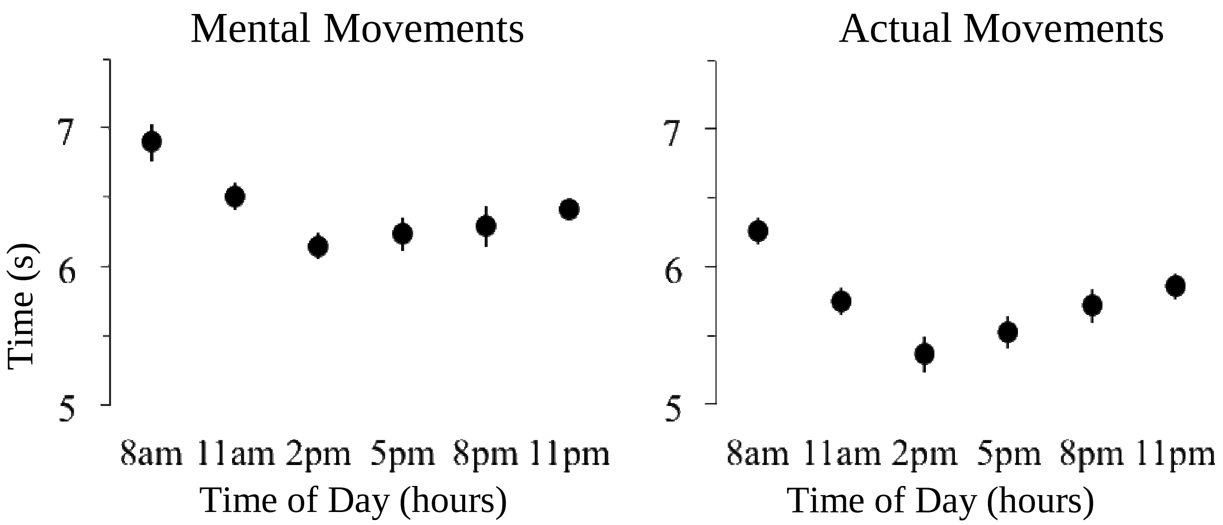

S 1. Mean values and SE for mental and actual movement times across subjects during the day.
